# A Rare Course of Chiari Malformation With Large Syringomyelia Presenting at 54 Years Old

**DOI:** 10.7759/cureus.78399

**Published:** 2025-02-02

**Authors:** Masato Tanaka, Sneha Sharma, Kushal H Gori, Md Shohidullah, Koji Uotani

**Affiliations:** 1 Orthopedic Surgery, Okayama Rosai Hospital, Okayama, JPN; 2 Orthopedic Surgery, Okayama University Hospital, Okayama, JPN; 3 Orthopedics, North Delhi Municipal Corporation (DMC) Medical College, New Delhi, IND

**Keywords:** chiari malformation, foramen magnum decompression, large syringomyelia, navigation system, syringosubarachnoid shunting

## Abstract

Chiari malformation type 1 (CM1) is considered a congenital condition. The symptoms include severe headache, hypalgesia, and loss of temperature sensation. It constitutes a significant burden among children and young adults. The onset of symptoms of CM1 is more commonly observed in relatively young children and is very rare in those over 50 years old. This study aims to present a rare surgical case of CM1 associated with a large syringomyelia in a 54-year-old man.

A 54-year-old man with low back pain was introduced to our department. He had slight hyperreflexia of the extremities, slight muscle weakness in both legs, and numbness in the right leg (3/10). He also had urinary and bowel incontinence and spastic gait. Cervical magnetic resonance imaging (MRI) showed CM1 with large syringomyelia extending from C1 to T11. The cervical canal was widened because of a long history of spinal cord expansion.

The patient was successfully treated surgically by foramen magnum decompression and syringosubarachnoid shunting under the guidance of O-arm navigation. The muscle weakness and sensory function recovered almost entirely on the one-year follow-up. The patient's cervical Japanese Orthopedic Association (JOA) score had improved from 11/17 to 16/17.

Gradually enlarging syringomyelia with slight CM1 is rare, but surgeons should consider this condition's possibility. Foramen magnum decompression achieves good results even in cases with a long history of syringomyelia. This new navigation technique provides an excellent result for a large syringomyelia with CM1.

## Introduction

An Austrian pathologist, Hans Chiari, reported Chiari's malformation in 1891 [1]. There are several criteria for Chiari malformation type 1 (CM1), but the most common one is the distal displacement >4 mm of the cerebellar tonsils below the foramen magnum. The incidence of CM1 in the pediatric population is about 2% [2]. Chiari malformation type 2 (CM2) is defined as the distal displacement of the cerebellum, fourth ventricle, and medulla. In Chiari malformation type 3 (CM3), there is a defect in the back of the head or neck as well as a form of dysraphism with a portion of the cerebellum and/or brainstem pushing through it. Chiari malformation type 4 (CM4) is the most severe form and the rarest, where the cerebellum does not fully develop and parts of it are missing. Parts of the skull and spinal cord may also be visible. This type is generally incompatible with life. Chiari malformation type 5 (CM5) is characterized by cerebellar aplasia and occipital lobe herniation [3].

For CM1, spinal cord syrinx is one of the most common comorbidities and has a reported occurrence of between 23% and 80% [4]. Most patients with CM1 remained asymptomatic [5]. However, surgical intervention is indicated if the patient has severe symptoms such as cough-associated severe headaches and objective abnormal neurological findings [6]. Among several surgical techniques, foramen magnum decompression (FMD) is the most popular and safe procedure, with an 83% success rate [7]. The onset of symptoms of CM1 is more commonly observed in relatively young children and is very rare in those over 50 years old. In this report, we present a rare surgical case of CM1 associated with a large syringomyelia in a 54-year-old man.

Approval was obtained from the Institutional Review Boards at Okayama Rosai Hospital (approval number: 433). This study was conducted according to the Declaration of Helsinki guidelines and approved on September 2023. Also, the patient provided the necessary consent.

## Case presentation

A 54-year-old man with lumbago was introduced to our department. In the examination, he had slight hyperreflexia of the extremities, slight muscle weakness in both legs (Manual Muscle Testing (MMT) 4), and numbness (3/10) in the right leg. He also had urinary and bowel incontinence and spastic gait. The patient's cervical Japanese Orthopedic Association (JOA) score was 11/17.

**Figure 1:**
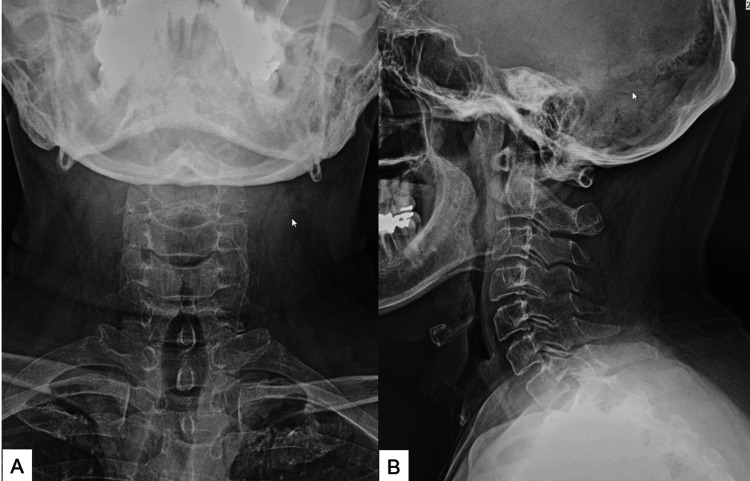
Preoperative roentgenogram: (A) anteroposterior roentgenogram and (B) lateral flexion roentgenogram. The sagittal canal width was increased at the C5 level (20.3 mm).

Preoperative cervical roentgenograms showed no deformity, though the anteroposterior width of the cervical canal was increased in lateral view (Figure 1).

**Figure 2:**
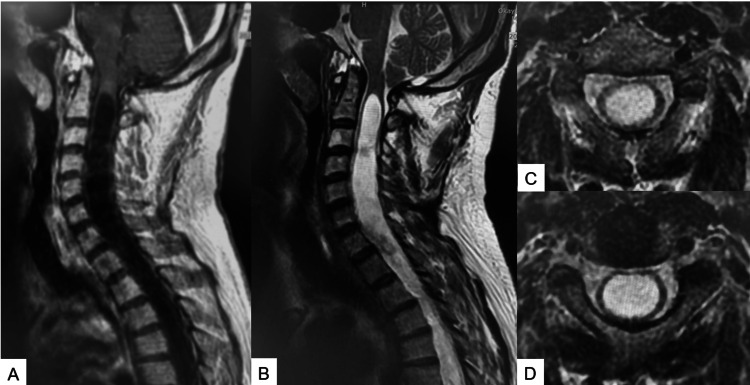
Preoperative MRI: (A) T1 sagittal MRI, (B) T2 sagittal MRI, (C) T2 axial MRI at C4, and (D) T2 axial MRI at C5/6. MRI: magnetic resonance imaging

Preoperative magnetic resonance imaging (MRI) indicated a 7 mm downward herniation of the cerebellar tonsils through the foramen magnum (Figure 2).

**Figure 3:**
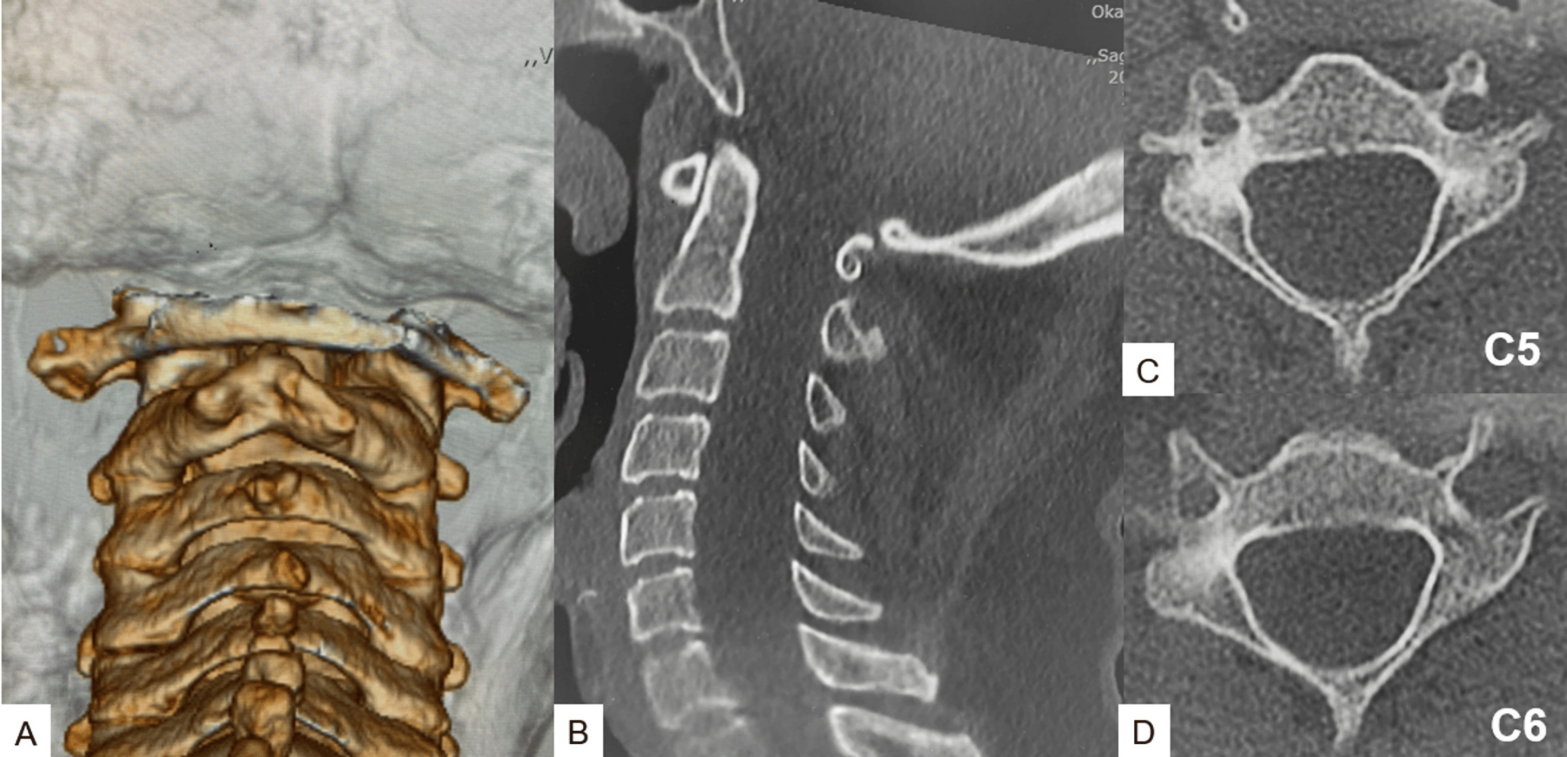
Preoperative CT: (A) posterior aspect of 3D reconstruction CT, (B) mid-sagittal reconstruction, (C) axial section at C5, and (D) axial section at C5. An enlarged spinal canal and bony scalloping can be seen. CT: computed tomography

Computed tomography (CT) showed scalloping of cervical laminae (Figure 3). The cervical canal was widened because of a long history of spinal cord expansion.

**Figure 4:**
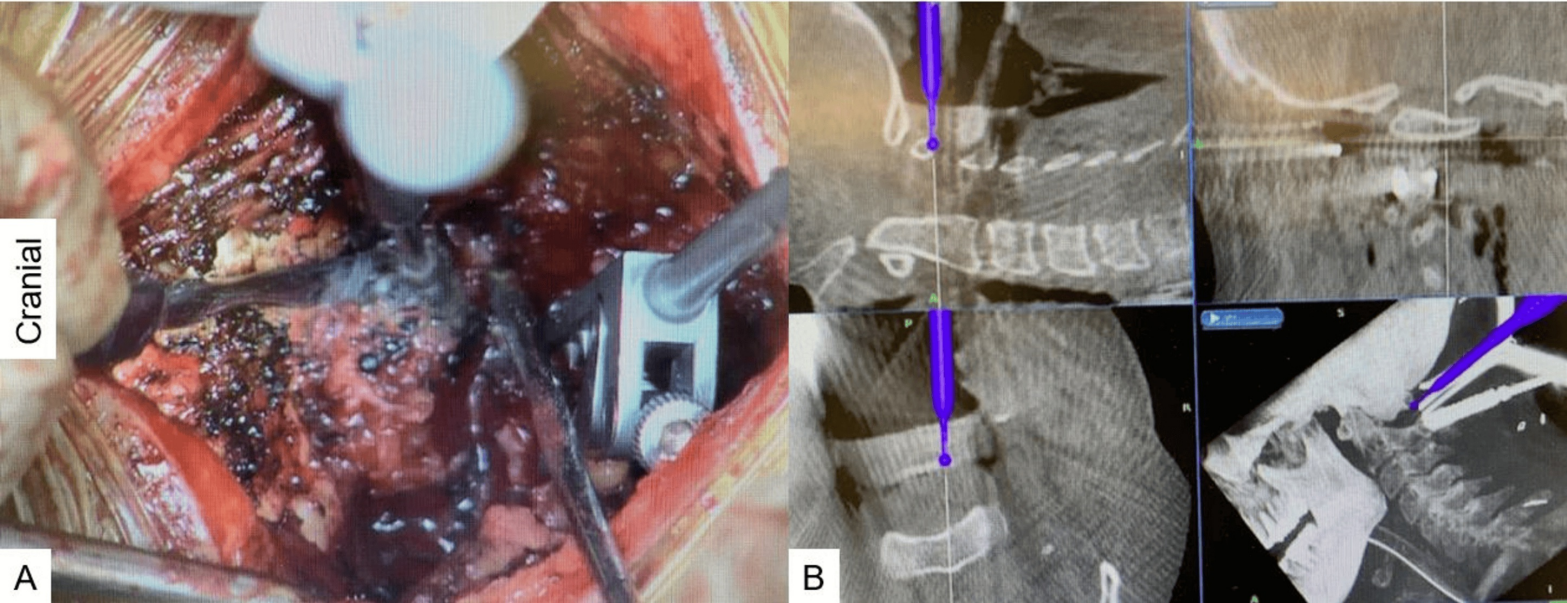
Posterior arch resection: (A) intraoperative image and (B) navigation monitor. The navigated high-speed burr was used to make a precise location of the gutter.

**Figure 5:**
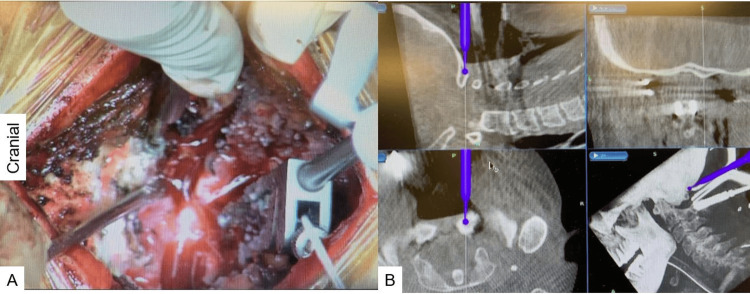
FMD: (A) intraoperative image and (B) navigation monitor. Adequate decompression was performed with the navigated high-speed burr. FMD: foramen magnum decompression

The patient underwent FMD under the guidance of O-arm navigation. He was in a prone position with the neck in a flexed position on a full carbon frame with a skull clamp. The procedure was performed under neuromonitoring. The occiput, atlas, and axis were exposed with a posterior midline incision. First, a reference frame was applied to the C2. Then, the O-arm was positioned, and 3D reconstructed CT images were taken. After verifying the spinal instrument to the navigation, the C1 posterior arch was removed with the help of navigated high-speed burr. A 3-cm-wide suboccipital craniectomy around the foramen magnum was performed under navigation guidance. The occipital bone's width and the foramen magnum's exact location were easily identified by navigation (Figure 4 and Figure 5).

The patient was successfully treated surgically. FMD was performed under the guidance of O-arm navigation. The superficial layer of the dura was resected, and the deep layer of the dura was enlarged. The surgery took 139 minutes, and the intraoperative blood loss was 180 ml. The patient had no postoperative complications.

**Figure 6:**
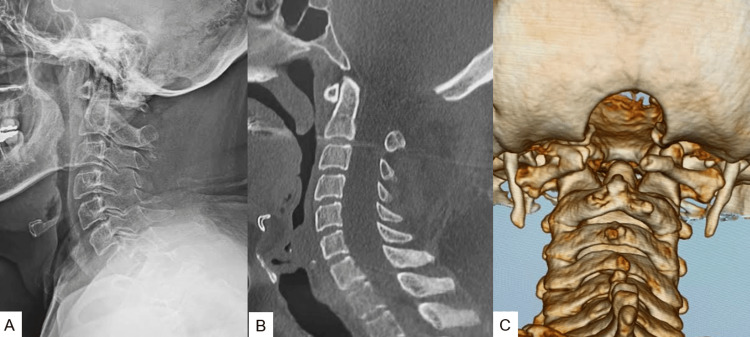
Postoperative images: (A) lateral radiogram, (B) mid-sagittal reconstruction CT, and (C) 3D CT. The foramen magnum was adequately decompressed. CT: computed tomography

Postoperative CT showed an adequate bony resection (Figure 6).

**Figure 7:**
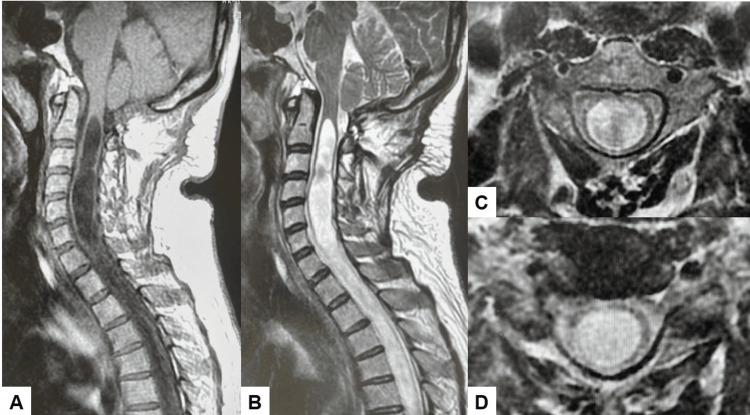
MRI after FMD: (A) T1 sagittal MRI, (B) T2 sagittal MRI, (C) T2 axial MRI at C4, and (D) T2 axial MRI at C5/6. The large syrinx was a little decreased. MRI: magnetic resonance imaging; FMD: foramen magnum decompression

The postoperative MRI of FMD indicated a slightly reduced syringomyelia (Figure 7).

**Figure 8:**
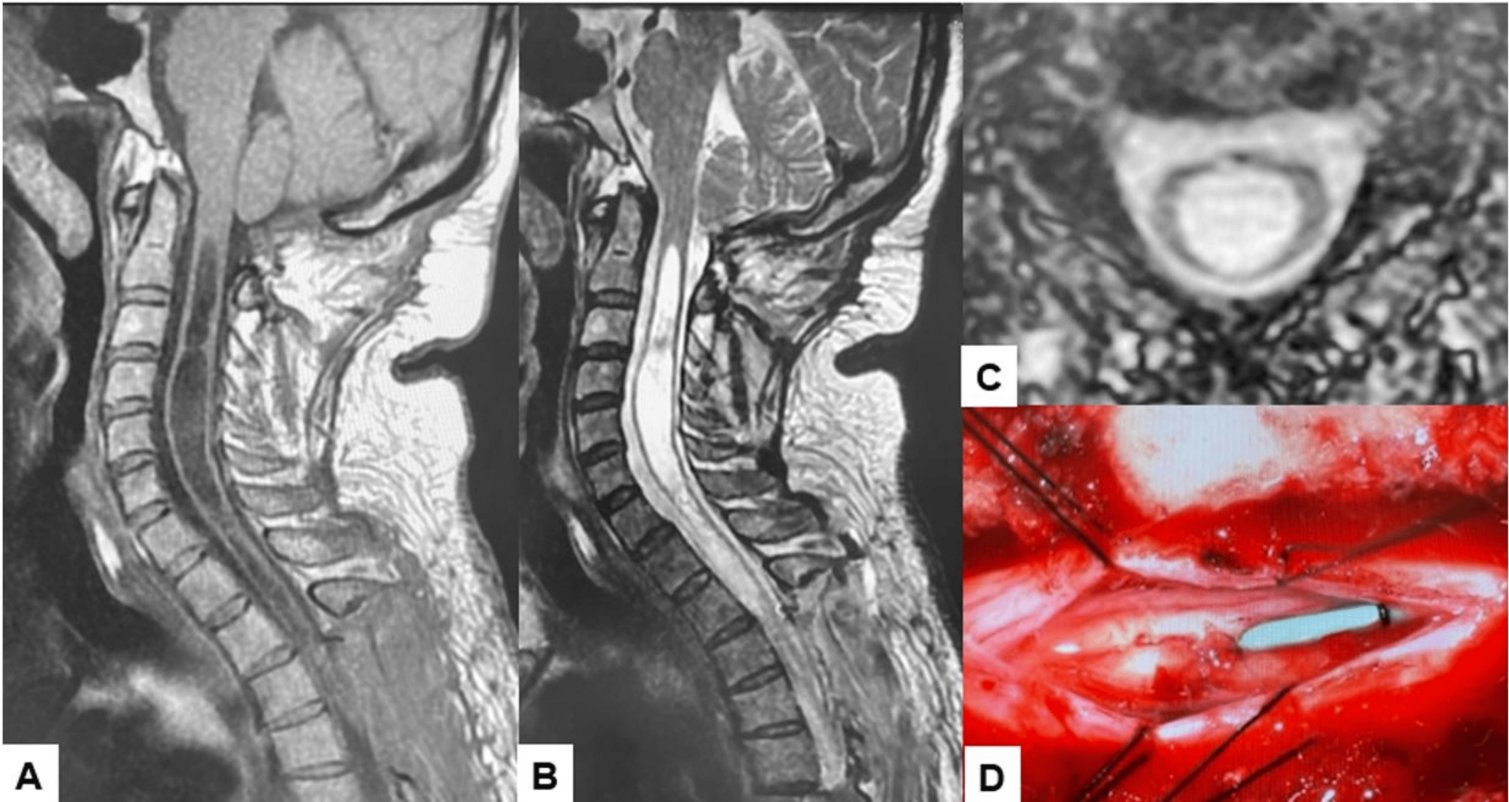
Images after SS shunting: (A) T1 sagittal MRI, (B) T2 sagittal MRI, (C) T2 axial MRI at C4, and (D) SS shunting. The syringomyelia showed a decrease in morphology. MRI: magnetic resonance imaging; SS: syringosubarachnoid

The patient's symptoms remained severe after six months of observation, so syringosubarachnoid (SS) shunting was performed (Figure 8).

At 12 months follow-up, the patient recovered almost entirely well (JOA 16/17). He had no neck pain but slight numbness in his bilateral hands. His urinary and bowel functions were recovered.

## Discussion

CM1 is a condition commonly present in the general population with an incidence of 0.5-3.5% [8]. A widely accepted theory is the underdevelopment of the posterior fossa, which results in its overcrowding and the downward displacement of cerebellar tonsils, blocking the flow of the cerebrospinal fluid. Recently, another theory proposed by Goel et al. states that the underlying cause of CM1 symptoms is atlantoaxial joint instability and cerebellar tonsil herniation is a protective measure against mechanical pinching. This theory does not apply to asymptomatic CM1 patients [9]. Wan et al., in a morphometric study on 47 adult CM1 patients, did a high-resolution CT of the atlanto-occipital joint and observed that symptomatic CM1 patients have an anomaly of occipital condyles and facets on the atlas [10].

The symptoms can be highly variable and not unique to CM1, making the diagnosis difficult [11,12]. In adults, symptoms include occipital headaches induced by cough or the Valsalva maneuver [13]. Cerebellar syndromes like truncal and appendicular ataxia can also be present [12]. Though symptom onset is at any age, it usually manifests in the 20s or 30s. Around 60-85% of CM1 cases are associated with syringomyelia [14]. 

Diagnosis and management of CM1 in adults approaching old age are often delayed. Clinical suspicion is essential in reaching a diagnosis. A thorough history-taking and clinical examination followed by an MRI should be done in patients suspected of having CM1. In a review study conducted by Arnautovic et al. in 2015 on literature published over 48 years (1965-2013), including 40 series of adult patients and a combined series of 61 reports of adult and pediatric patients, the mean age of presentation was 41 years with the peak age of presentation being 41 years and the next peak age 46 years [8]. The patient in the present study is a 54-year-old man, and this data will help physicians focus on the diagnosis in symptomatic patients of this age group as well.

Although there is an ongoing controversy about the optimum surgical technique to be used in the treatment of CM1, the main goal of surgery is to relieve the craniospinal pressure dissociation by decreasing the compression at the cervicomedullary junction, which causes the reduction in the size of syrinx [15]. The most commonly used technique is FMD, which increases the space for the cerebellum and relieves pressure on the spinal cord [16]. Many adjunctive techniques include lysing the epidural membranes, duraplasty, arachnoid membrane release, and obex plugging [15]. In our case of a large syringomyelia, these techniques should have been considered in the first surgery. FMD combined with posterior arch resection and duraplasty is a commonly accepted surgery [17]. A crucial aspect of the surgery is determining the extent of the bony decompression. A smaller extent of craniectomy may not be sufficient to relieve the compression, whereas a larger one carries a risk of descent of the cerebellum through the defect created [15].

We used navigation to locate the area and extent of decompression accurately. It also guides the high-speed burr providing real-time intraoperative guidance and keeping clear important anatomic structures in a relatively small working area. The commonly found distorted anatomy of the cranial-cervical junction and associations like platybasia and hypoplasia of basic occiput make the surgery challenging. The use of a navigation system reduces the risk of iatrogenic complications in such cases [18].

An SS or syringopleural (SP) shunt can be placed in patients with no response to this treatment [19]. According to a systemic review of 473 cases of these shunting, the rate of clinical improvement was estimated as 61% for SS shunting and 64% for SP shunting. However, the incidences of revision surgery for SS shunting and SP shunting were 13% and 28%, respectively [20]. In our case, the patient had a good recovery of neurological status and no revision surgery.

This report's limitation is that the follow-up period is relatively short. The follow-up MRI indicated that a large syringomyelia still existed.

## Conclusions

Gradually enlarged syringomyelia with slight CM1 is rare. This syringomyelia with CM1 may become symptomatic in relatively old age, but surgeons should consider this possibility. FMD (tonsils decompression) with navigation for CM1 is usually beneficial in reducing the radiation hazard. However, SS shunting may be necessary for a large syringomyelia.
